# Longitudinal neurodegeneration in Early‐Onset Alzheimer’s Disease: A summary of MRI‐derived atrophy in LEADS

**DOI:** 10.1002/alz.091402

**Published:** 2025-01-09

**Authors:** Alexandra Touroutoglou, Yuta Katsumi, Ryan Eckbo, Michael Brickhouse, Ani Eloyan, Kelly N. Nudelman, Tatiana M. Foroud, Jeffrey L. Dage, Maria C. Carrillo, Gil D. Rabinovici, Liana G. Apostolova, Bradford C. Dickerson, the LEADS Consortium

**Affiliations:** ^1^ Frontotemporal Disorders Unit and Massachusetts Alzheimer’s Disease Research Center, Department of Neurology, Massachusetts General Hospital and Harvard Medical School, Boston, MA USA; ^2^ Massachusetts General Hospital and Harvard Medical School, Boston, MA USA; ^3^ Department of Biostatistics, Brown University, Providence, RI USA; ^4^ Department of Medical and Molecular Genetics, Indiana University School of Medicine, Indianapolis, IN USA; ^5^ Department of Neurology, Indiana University School of Medicine, Indianapolis, IN USA; ^6^ Alzheimer's Association, Chicago, IL USA; ^7^ Department of Neurology, Memory and Aging Center, University of California San Francisco, San Francisco, CA USA; ^8^ Memory and Aging Center, Weill Institute for Neurosciences, University of California, San Francisco, San Francisco, CA USA; ^9^ Department of Radiology and Imaging Sciences, Indiana University School of Medicine, Indianapolis, IN USA

## Abstract

**Background:**

Prior work has advanced our understanding of cortical atrophy in early‐onset Alzheimer’s disease (EOAD), but longitudinal data are sparse. Current longitudinal MRI studies point to progressive atrophy in cerebral cortex exhibiting a posterior‐to‐anterior gradient, but these studies include small samples with mostly amnestic EOAD. Here, we analyzed a large sample of sporadic EOAD patients from the Longitudinal Early‐Onset Alzheimer's Disease Study (LEADS) to test the central hypothesis that areas in our recently described EOAD signature (Touroutoglou et al., 2023) affected at baseline in the posterior lateral temporal cortex, inferior parietal lobule, and PCC/precuneus will continue to degenerate and additional longitudinal atrophy will be found in the medial temporal lobe and frontal regions as cognitive decline progresses over time in multiple domains.

**Method:**

We investigated longitudinal changes in cortical thickness by analyzing structural MRI data collected from 367 patients with EOAD and 99 cognitively unimpaired (CN) older adults, totaling 839 MRI scans across the cohorts with up to 4 years of follow‐up. MRI data were longitudinally processed in FreeSurfer 6.0. Linear mixed effects models were constructed to estimate the rate of cortical atrophy with random intercepts and slopes for individual participants while controlling for baseline age and sex.

**Result:**

EOAD patients exhibited cortical atrophy at a faster rate than controls in widespread areas of the cerebral cortex. As expected, the regions exhibiting accelerated longitudinal atrophy included not only the EOAD signature regions as a whole (EOAD: ‐0.052±0.002 mm/year vs. CN: 0.0001±0.002 mm/year; Dslopes = ‐0.052, p<.001), but also those that were minimally atrophied at baseline, such as superior frontal gyrus (EOAD: ‐0.052+/‐0.004 vs. CN: ‐0.001+/‐0.004, Dslopes = ‐ 0.051, p<.001) and medial temporal lobe (EOAD: ‐0.083±0.005 mm/year vs. CN: 0.001±0.006 mm/year; Dslopes = ‐0.082, p<.001). We observed no difference in the rate of atrophy in the calcarine fissure (a control region not expected to change; Dslopes = ‐0.002, p£.69).

**Conclusion:**

Our findings show that neurodegeneration in EOAD accelerates over time in the EOAD signature regions and spreads to additional areas within large‐scale brain networks (consistent with those observed in late‐onset AD) contributing to the worsening of symptoms over time.

